# Supplementary material for: Research on multi-agent genetic algorithm based on tabu search for the job shop scheduling problem
Source: PLoS One. 2019 Sep 27;14(9):e0223182. doi: 10.1371/journal.pone.0223182 (PMC6764687; doi:10.1371/journal.pone.0223182)
Supplement: S1 File — (DOCX) [file pone.0223182.s001.docx]

# FT06 6×6

Machine_Matrix:

{2,0,1,3,5,4},{1,2,4,5,0,3},{2,3,5,0,1,4},{1,0,2,3,4,5},{2,1,4,5,0,3},{1,3,5,0,4,2}

Time_Matrix:

{1,3,6,7,3,6},{8,5,10,10,10,4},{5,4,8,9,1,7},{5,5,5,3,8,9},{9,3,5,4,3,1},{3,3,9,10,4,1}

# FT10 10×10

Machine_Matrix:

{0,1,2,3,4,5,6,7,8,9},{0,2,4,9,3,1,6,5,7,8},{1,0,3,2,8,5,7,6,9,4},{1,2,0,4,6,8,7,3,9,5},{2,0,1,5,3,4,8,7,9,6},

{2,1,5,3,8,9,0,6,4,7},{1,0,3,2,6,5,9,8,7,4},{2,0,1,5,4,6,8,9,7,3},{0,1,3,5,2,9,6,7,4,8},{1,0,2,6,8,9,5,3,4,7}

Time_Matrix:

{29,78,9,36,49,11,62,56,44,21},{43,90,75,11,69,28,46,46,72,30},{91,85,39,74,90,10,12,89,45,33},{81,95,71,99,9,52,85,98,22,43},{14,6,22,61,26,69,21,49,72 ,53},{84,2,52,95,48,72,47,65,6,25}{46,37,61,13,32,21,32,89,30,55},{31,86,46,74,32,88,19,48,36,79},{76,69,76,51,85,11,40,89,26,74},{85,13,61,7,64,76,47,52,90,45}

# FT20 20×5

Machine_Matrix:

{0,1,2,3,4},{0,1,3,2,4},{1,0,2,4,3},{1,0,4,2,3},{2,1,0,3,4},

{2,1,4,0,3},{1,0,2,3,4},{2,1,0,3,4},{0,3,2,1,4},{1,2,0,3,4},

{1,3,0,4,2},{2,0,1,3,4},{0,2,1,3,4},{2,0,1,3,4},{0,1,4,2,3},

{1,0,3,4,2},{0,2,1,3,4},{0,1,4,2,3},{1,2,0,3,4},{0,1,2,3,4}

Time_Matrix:

{29,9,49,62,44},{43,75,69,46,72},{91,39,90,12,45},{81,71,9,85,22},

{14,22,26,21,72},{84,52,48,47,6},{46,61,32,32,30},{31,46,32,19,36},

{76,76,85,40,26},{85,61,64,47,90},{78,36,11,56,21},{90,11,28,46,30},

{85,74,10,89,33},{95,99,52,98,43},{6,61,69,49,53},{2,95,72,65,25},

{37,13,21,89,55},{86,74,88,48,79},{69,51,11,89,74},{13,7,76,52,45}

# LA01 10×5

Machine_Matrix:

{1,0,4,3,2},{0,3,4,2,1},{3,4,1,2,0},{1,0,4,2,3},{0,3,2,1,4},

{1,2,4,0,3},{3,4,1,2,0},{2,0,1,3,4},{3,1,4,0,2},{4,3,2,1,0}

Time_Matrix:

{21,53,95,55,34},{21,52,16,26,71},{39,98,42,31,12},{77,55,79,66,77},

{83,34,64,19,37},{54,43,79,92,62},{69,77,87,87,93},{38,60,41,24,83},

{17,49,25,44,98},{77,79,43,75,96}

# LA02 10×5

Machine_Matrix:

{0,3,1,4,2},{4,2,0,1,3},{1,2,4,0,3},{2,1,4,0,3},{4,0,3,2,1},

{1,0,4,3,2},{4,1,3,0,2},{1,0,2,3,4},{4,0,2,1,3},{4,2,1,3,0}

Time_Matrix:

{20,87,31,76,17},{25,32,24,18,81},{72,23,28,58,99},{86,76,97,45,90},

{27,42,48,17,46},{67,98,48,27,62},{28,12,19,80,50},{63,94,98,50,80},

{14,75,50,41,55},{72,18,37,79,61}

# LA03 10×5

Machine_Matrix:

{1,2,0,4,3},{2,1,0,4,3},{2,3,4,0,1},{4,0,2,1,3},{4,0,1,3,2},

{4,0,1,2,3},{3,2,0,4,1},{4,1,0,2,3},{4,0,3,2,1},{4,1,0,2,3}

Time_Matrix:

{23,45,82,84,38},{21,29,18,41,50},{38,54,16,52,52},{37,54,74,62,57},

{57,81,61,68,30},{81,79,89,89,11}{33,20,91,20,66},{24,84,32,55,8},

{56,7,54,64,39},{40,83,19,8,7}

# LA04 10×5

Machine_Matrix:

{0,2,3,4,1},{1,3,4,2,0},{1,0,3,4,2},{2,4,0,3,1},{1,3,4,0,2},

{3,2,0,4,1},{2,1,0,3,4},{1,3,0,4,2},{2,4,0,1,3},{2,4,3,1,0}

Time_Matrix:

{12,94,92,91,7},{19,11,66,21,87},{14,75,13,16,20},{95,66,7,7,77},{45,6,89,15,34},

{77,20,76,88,53},{74,88,52,27,9},{88,69,62,98,52},{61,9,62,52,90},{54,5,59,15,88}

# LA05 10×5

Machine_Matrix:

{1,0,4,2,3},{4,3,0,2,1},{1,3,2,0,4},{0,3,4,1,2},{4,2,3,1,0},

{3,0,4,1,2},{0,3,1,4,2},{4,2,3,1,0},{2,3,1,0,4},{2,3,0,4,1}

Time_Matrix:

{72,87,95,66,60},{5,35,48,39,54},{46,20,21,97,55},{59,19,46,34,37},

{23,73,25,24,28},{28,45,5,78,83},{53,71,37,29,12},{12,87,33,55,38},

{49,83,40,48,7},{65,17,90,27,23}

# LA06 15×5

Machine_Matrix:

{1,2,4,0,3},{3,4,1,2,0},{2,0,1,3,4},{3,1,4,0,2},{4,3,2,1,0},

{2,1,0,3,4},{0,3,1,4,2},{0,1,2,4,3},{2,3,4,0,1},{0,4,3,1,2},

{4,2,0,3,1},{0,4,2,1,3},{4,3,1,2,0},{4,1,0,2,3},{0,1,2,4,3}

Time_Matrix:

{21,34,95,53,55},{52,16,71,26,21},{31,12,42,39,98},{77,77,79,55,66},

{37,34,64,19,83},{43,54,92,62,79},{93,69,87,77,87},{60,41,38,83,24},

{98,17,25,44,49},{96,77,79,75,43},{28,35,95,76,7},{61,10,95,9,35},

{59,16,91,59,46},{43,52,28,27,50},{87,45,39,9,41}

# LA07 15×5

Machine_Matrix:

{0,4,1,3,2},{0,1,4,3,2},{3,0,2,1,4},{0,1,4,3,2},{3,1,0,2,4},

{1,2,0,4,3},{2,1,0,4,3},{2,3,4,0,1},{4,0,2,1,3},{4,0,1,3,2},

{4,0,1,2,3},{3,2,0,4,1},{4,1,0,2,3},{4,0,3,2,1},{4,1,0,2,3}

Time_Matrix:

{47,57,71,96,14},{75,60,22,79,65},{32,33,69,31,58},{44,34,51,58,47},

{29,44,62,17,8},{15,40,97,38,66},{58,39,57,20,50},{57,32,87,63,21},

{56,84,90,85,61},{15,20,67,30,70},{84,82,23,45,38},{50,21,18,41,29},

{16,52,52,38,54},{37,54,57,74,62},{57,61,81,30,68}

# LA08 15×5

Machine_Matrix:

{3,2,0,4,1},{2,1,0,3,4},{1,3,0,4,2},{2,4,0,1,3},{2,4,3,1,0},

{4,3,2,1,0},{4,3,0,1,2},{3,2,0,1,4},{3,0,4,2,1},{4,2,3,0,1},

{0,1,4,3,2},{0,4,2,3,1},{0,3,4,2,1},{3,1,0,4,2},{2,0,3,1,4}

Time_Matrix:

{92,94,12,91,7},{21,19,87,11,66},{14,13,75,16,20},{95,66,7,77,7},{34,89,6,45,15},

{88,77,20,53,76},{9,27,52,88,74},{69,52,62,88,98},{90,62,9,61,52},{5,54,59,88,15},

{41,50,78,53,23},{38,72,91,68,71},{45,95,52,25,6},{30,66,23,36,17},{95,71,76,8,88}

# LA09 15×5

Machine_Matrix:

{1,3,2,0,4},{3,1,2,4,0},{4,3,1,2,0},{0,1,2,3,4},{0,4,2,3,1},

{0,3,1,2,4},{3,2,4,1,0},{2,1,3,4,0},{2,4,0,1,3},{2,4,3,1,0},

{4,3,2,0,1},{1,0,3,4,2},{4,3,0,1,2},{3,1,2,0,4},{0,1,2,4,3}

Time_Matrix:

{66,85,84,62,19},{59,64,46,13,25},{88,80,73,53,41},{14,67,57,74,47},

{84,64,41,84,78},{63,28,46,26,52},{10,17,73,11,64},{67,97,95,38,85},

{95,46,59,65,93},{43,85,32,85,60},{49,41,61,66,90},{17,23,70,99,49},

{40,73,73,98,68},{57,9,7,13,98},{37,85,17,79,41}

# LA10 15×5

Machine_Matrix:

{1,2,3,0,4},{1,0,4,3,2},{0,1,2,4,3},{3,1,2,0,4},{2,0,1,3,4},

{3,4,2,0,1},{1,4,0,2,3},{2,3,1,4,0},{0,3,4,1,2},{2,4,3,0,1},

{0,4,3,2,1},{2,0,1,4,3},{3,2,1,4,0},{1,2,4,0,3},{3,2,0,4,1}

Time_Matrix:

{58,44,5,9,58},{89,97,96,77,84},{77,87,81,39,85},{57,21,31,15,73},{48,40,49,70,71},

{34,82,80,10,22},{91,75,55,17,7},{62,47,72,35,11},{64,75,50,90,94},{67,20,15,12,71}

{52,93,68,29,57},{70,58,93,7,77},{27,82,63,6,95},{87,56,36,26,48},{76,36,36,15,8}

# LA11 20×5

Machine_Matrix:

{2,1,0,3,4},{0,3,1,4,2},{0,1,2,4,3},{2,3,4,0,1},{0,4,3,1,2},

{4,2,0,3,1},{0,4,2,1,3},{4,3,1,2,0},{4,1,0,2,3},{0,1,2,4,3},

{0,3,1,4,2},{4,2,0,1,3},{1,2,4,0,3},{2,1,4,0,3},{4,0,3,2,1},

{1,0,4,3,2},{4,1,3,0,2},{1,0,2,3,4},{4,0,2,1,3},{4,2,1,3,0}

Time_Matrix:

{34,21,53,55,95},{21,52,71,16,26},{12,42,31,98,39},{66,77,79,55,77},

{83,37,34,19,64},{79,43,92,62,54},{93,77,87,87,69},{83,24,41,38,60},

{25,49,44,98,17},{96,75,43,77,79},{95,76,7,28,35},{10,95,61,9,35},

{91,59,59,46,16},{27,52,43,28,50},{9,87,41,39,45},{54,20,43,14,71},

{33,28,26,78,37},{89,33,8,66,42},{84,69,94,74,27},{81,45,78,69,96}

# LA12 20×5

Machine_Matrix:

{1,0,4,2,3},{3,4,1,0,2},{4,3,1,2,0},{1,3,4,2,0},{3,1,2,0,4},

{1,2,3,0,4},{1,0,3,4,2},{3,4,2,0,1},{0,2,1,4,3},{0,4,3,2,1},

{0,2,3,4,1},{1,3,4,2,0},{1,0,3,4,2},{2,4,0,3,1},{1,3,4,0,2},

{3,2,0,4,1},{2,1,0,3,4},{1,3,0,4,2},{2,4,0,1,3},{2,4,3,1,0}

Time_Matrix:

{23,82,84,45,38},{50,41,29,18,21},{16,54,52,38,52},{62,57,37,74,54},

{68,61,30,81,57},{89,89,11,79,81},{66,91,33,20,20},{8,24,55,32,84},

{7,64,39,56,54},{19,40,7,8,83},{63,64,91,40,6},{42,61,15,98,74},

{80,26,75,6,87},{39,22,75,24,44},{15,79,8,12,20},{26,43,80,22,61},

{62,36,63,96,40},{33,18,22,5,10},{64,64,89,96,95},{18,23,15,38,8}

# LA13 20×5

Machine_Matrix:

{3,0,1,4,2},{1,0,2,3,4},{3,1,0,2,4},{2,0,3,1,4},{2,3,1,0,4},

{1,3,2,0,4},{3,1,2,4,0},{4,3,1,2,0},{0,1,2,3,4},{0,4,2,3,1},

{0,3,1,2,4},{3,2,4,1,0},{2,1,3,4,0},{2,4,0,1,3},{2,4,3,1,0},

{4,3,2,0,1},{1,0,3,4,2},{4,3,0,1,2},{3,1,2,0,4},{0,1,2,4,3}

Time_Matrix:

{60,87,72,95,66},{54,48,39,35,5},{20,46,97,21,55},{37,59,19,34,46},

{73,25,24,28,23},{78,28,83,45,5},{71,37,12,29,53},{12,33,55,87,38},

{48,40,49,83,7},{90,27,65,17,23},{62,85,66,84,19},{59,46,13,64,25},

{53,73,80,88,41},{57,47,14,67,74},{41,64,84,78,84},{52,28,26,63,46},

{11,64,10,73,17},{38,95,85,97,67},{93,65,95,59,46},{60,85,43,85,32}

# LA14 20×5

Machine_Matrix:

{3,4,2,0,1},{1,4,0,2,3},{2,3,1,4,0},{0,3,4,1,2},{2,4,3,0,1},

{0,4,3,2,1},{2,0,1,4,3},{3,2,1,4,0},{1,2,4,0,3},{3,2,0,4,1},

{4,2,0,1,3},{3,1,0,2,4},{1,3,0,4,2},{4,0,3,2,1},{2,1,4,3,0},

{4,1,3,0,2},{1,0,4,3,2},{0,4,2,1,3},{1,4,0,3,2},{3,1,0,2,4}

Time_Matrix:

{5,58,44,9,58},{89,96,97,84,77},{81,85,87,39,77},{15,57,73,21,31},

{48,71,70,40,49},{10,82,34,80,22},{17,55,91,75,7},{47,62,72,35,11},

{90,94,50,64,75},{15,67,12,20,71},{93,29,52,57,68},{77,93,58,70,7},

{63,27,95,6,82},{36,26,48,56,87},{36,8,15,76,36},{78,84,41,30,76},

{78,75,88,13,81},{54,40,13,82,29},{26,82,52,6,6},{54,64,54,32,88}

# LA15 20×5

Machine_Matrix:

{0,2,1,3,4},{2,3,0,4,1},{1,4,2,3,0},{2,4,0,3,1},{2,0,1,3,4},

{0,4,1,3,2},{4,3,1,2,0},{0,2,1,4,3},{4,0,3,2,1},{1,0,4,2,3},

{0,1,2,4,3},{2,0,3,1,4},{0,2,1,3,4},{0,3,2,1,4},{1,0,4,3,2},

{1,2,4,0,3},{1,4,2,0,3},{3,0,2,4,1},{0,1,2,3,4},{1,2,4,0,3}

Time_Matrix:

{6,40,81,37,19},{40,32,55,81,9},{46,65,70,55,77},{21,65,64,25,15},

{85,40,44,24,37},{89,29,83,31,84},{59,38,80,30,8},{80,56,77,41,97},

{56,91,50,71,17},{40,88,59,7,80},{45,29,8,77,58},{36,54,96,9,10},

{28,73,98,92,87},{70,86,27,99,96},{95,59,56,85,41},{81,92,32,52,39},

{7,22,12,88,60},{45,93,69,49,27},{21,84,61,68,26},{82,33,71,99,44}

# LA16 10×10

Machine_Matrix:

{1,6,9,8,7,2,0,4,3,5},{4,2,5,9,0,7,1,8,6,3},{3,2,8,1,4,9,7,6,0,5},{1,3,2,7,8,9,6,0,5,4},

{2,0,5,6,7,1,4,9,3,8},{2,3,5,9,4,6,0,8,1,7},{3,2,0,1,9,8,6,5,4,7},{1,0,3,4,6,9,8,5,2,7},

{4,2,8,5,3,7,1,6,9,0},{8,9,2,4,3,0,7,6,1,5}

Time_Matrix:

{21,71,16,52,26,34,53,21,55,95},{55,31,98,79,12,66,42,77,77,39},

{34,64,62,19,92,79,43,54,83,37},{87,69,87,38,24,83,41,93,77,60},

{98,44,25,75,43,49,96,77,17,79},{35,76,28,10,61,9,95,35,7,95},

{16,59,46,91,43,50,52,59,28,27},{45,87,41,20,54,43,14,9,39,71},

{33,37,66,33,26,8,28,89,42,78},{69,81,94,96,27,69,45,78,74,84}

# LA17 10×10

Machine_Matrix:

{4,7,9,2,3,8,5,6,1,0},{8,5,1,7,2,3,6,9,4,0},{2,4,3,1,8,6,7,0,9,5},{0,8,3,7,5,2,4,6,1,9},

{9,0,4,8,6,2,5,3,7,1},{3,2,5,0,7,4,8,1,6,9},{1,7,8,3,4,5,6,0,2,9},{1,7,2,0,8,6,3,9,5,4},

{2,3,4,9,0,6,7,8,1,5},{1,0,5,3,9,7,8,2,6,4}

Time_Matrix:

{18,21,41,45,38,50,84,29,23,82},{57,16,52,74,38,54,62,37,54,52},

{30,79,68,61,11,89,89,81,81,57}{91,8,33,55,20,20,32,84,66,24},

{40,7,19,7,83,64,56,54,8,39},{91,64,40,63,98,74,61,6,42,15},

{80,39,24,75,75,6,44,26,87,22},{15,43,20,12,26,61,79,22,8,80},

{62,96,22,5,63,33,10,18,36,40},{96,89,64,95,23,18,15,64,38,8}

# LA18 10×10

Machine_Matrix:

{6,0,4,3,7,8,1,5,2,9},{3,9,6,5,0,8,4,2,7,1},{4,1,8,0,7,6,5,3,9,2},{9,1,4,3,8,2,6,0,7,5},

{3,2,6,9,7,0,4,5,1,8},{1,4,0,2,9,6,7,8,5,3},{1,3,0,2,9,7,8,4,6,5},{5,3,6,1,0,7,8,9,2,4},

{1,0,7,4,3,5,9,8,6,2},{4,8,2,3,1,6,7,9,5,0}

Time_Matrix:

{54,87,48,60,39,35,72,95,66,5},{20,46,34,55,97,19,59,21,37,46},

{45,24,28,28,83,78,23,25,5,73},{12,37,38,71,33,12,55,53,87,29},

{83,49,23,27,65,48,90,7,40,17},{66,25,62,84,13,64,46,59,19,85},

{73,80,41,53,47,57,74,14,67,88},{64,84,46,78,84,26,28,52,41,63},

{11,64,67,85,10,73,38,95,97,17},{60,32,95,93,65,85,43,85,46,59}

# LA19 10×10

Machine_Matrix:

{2,3,5,4,0,7,8,9,1,6},{4,7,1,8,0,3,2,5,9,6},{9,6,4,3,1,0,8,2,7,5},{1,2,7,5,8,4,3,6,9,0},

{6,1,3,0,2,8,4,7,9,5},{7,5,8,2,4,6,3,1,9,0},{6,1,4,5,2,3,7,8,9,0},{0,5,8,9,3,6,4,7,2,1},

{5,2,3,6,4,7,8,9,1,0},{9,4,6,7,0,2,8,5,3,1}

Time_Matrix:

{44,5,58,97,9,84,77,96,58,89},{15,31,87,57,77,85,81,39,73,21},

{82,22,10,70,49,40,34,48,80,71},{91,17,62,75,47,11,7,72,35,55},

{71,90,75,64,94,15,12,67,20,50},{70,93,77,29,58,93,68,57,7,52},

{87,63,26,6,82,27,56,48,36,95},{36,15,41,78,76,84,30,76,36,8},

{88,81,13,82,54,13,29,40,78,75},{88,54,64,32,52,6,54,82,6,26}

# LA20 10×10

Machine_Matrix:

{6,1,4,2,8,3,0,5,9,7},{7,2,9,4,1,5,8,0,3,6},

{2,5,0,3,1,6,4,8,7,9},{4,6,7,0,2,5,3,1,9,8},

{0,6,4,1,2,3,9,8,5,7},{2,6,3,5,1,8,0,9,4,7},

{4,3,1,5,6,7,8,9,0,2},{1,7,3,4,6,9,8,0,2,5},

{3,8,0,2,1,5,4,9,7,6},{0,2,3,5,6,9,8,4,7,1}

Time_Matrix:

{9,81,55,40,32,37,6,19,81,40},{21,70,65,64,46,65,25,77,55,15},

{85,37,40,24,44,83,89,31,84,29},{80,77,56,8,30,59,38,80,41,97},

{91,40,88,17,71,50,59,80,56,7},{8,9,58,77,29,96,45,10,54,36},

{70,92,98,87,99,27,86,96,28,73},{95,92,85,52,81,32,39,59,41,56},

{60,45,88,12,7,22,93,49,69,27},{21,61,68,26,82,71,44,99,33,84}

# LA21 15×10

Machine_Matrix:

{2,3,5,9,4,6,0,8,1,7},{3,2,0,1,9,8,6,5,4,7},{1,0,3,4,6,9,8,5,2,7},

{4,2,8,5,3,7,1,6,9,0},{8,9,2,4,3,0,7,6,1,5},{8,7,6,9,2,1,5,4,0,3},

{4,5,3,9,0,8,6,7,2,1},{5,4,2,6,1,7,0,3,9,8},{1,5,0,3,2,7,8,6,9,4},

{2,5,6,9,1,3,8,0,7,4},{1,4,0,2,9,8,5,3,7,6},{5,9,0,4,6,3,2,1,8,7},

{5,9,8,7,4,6,3,0,1,2},{1,8,0,2,9,3,5,6,4,7},{4,3,6,5,2,8,1,9,7,0}

Time_Matrix:

{34,55,95,16,21,71,53,52,21,26},{39,31,12,42,79,77,77,98,55,66},

{19,83,34,92,54,79,62,37,64,43},{60,87,24,77,69,38,87,41,83,93},

{79,77,98,96,17,44,43,75,49,25},{35,95,9,10,35,7,28,61,95,76},

{28,59,16,43,46,50,52,27,59,91},{9,20,39,54,45,71,87,41,43,14},

{28,33,78,26,37,8,66,89,42,33},{94,84,78,81,74,27,69,69,45,96},

{31,24,20,17,25,81,76,87,32,18},{28,97,58,45,76,99,23,72,90,86},

{27,48,27,62,98,67,48,42,46,17},{12,50,80,50,80,19,28,63,94,98},

{61,55,37,14,50,79,41,72,18,75}

# LA22 15×10

Machine_Matrix:

{9,5,4,2,7,3,1,0,8,6},{3,2,4,1,9,0,6,5,7,8},{8,7,2,0,9,5,6,3,1,4},

{3,2,6,4,7,8,5,9,0,1},{4,6,1,2,7,0,8,5,3,9},{6,0,4,3,7,8,1,5,2,9},

{3,9,6,5,0,8,4,2,7,1},{4,1,8,0,7,6,5,3,9,2},{9,1,4,3,8,2,6,0,7,5},

{3,2,6,9,7,0,4,5,1,8},{1,4,0,2,9,6,7,8,5,3},{1,3,0,2,9,7,8,4,6,5},

{5,3,6,1,0,7,8,9,2,4},{1,0,7,4,3,5,9,8,6,2},{4,8,2,3,1,6,7,9,5,0}

Time_Matrix:

{66,91,87,94,21,92,7,12,11,19},{13,20,7,14,66,75,77,16,95,7},

{77,20,34,15,88,89,53,6,45,76},{27,74,88,62,52,69,9,98,52,88},

{88,15,52,61,54,62,59,9,90,5},{71,41,38,53,91,68,50,78,23,72},

{95,36,66,52,45,30,23,25,17,6},{65,8,85,71,65,28,88,76,27,95},

{37,37,28,51,86,9,55,73,51,90},{39,15,83,44,53,16,46,24,25,82},

{72,48,87,66,5,54,39,35,95,60},{46,20,97,21,46,37,19,59,34,55},

{23,25,78,24,28,83,28,5,73,45},{37,53,87,38,71,29,12,33,55,12},

{90,17,49,83,40,23,65,27,7,48}

# LA23 15×10

Machine_Matrix:

{7,5,8,2,4,6,3,1,9,0},{6,1,4,5,2,3,7,8,9,0},{0,5,8,9,3,6,4,7,2,1},

{5,2,3,6,4,7,8,9,1,0}, {9,4,6,7,0,2,8,5,3,1},{6,5,1,7,8,4,0,2,9,3},

{7,0,8,4,2,1,9,3,6,5},{3,5,9,1,8,2,4,6,0,7},{0,7,2,8,4,6,5,1,9,3},

{2,1,7,6,4,0,5,3,9,8},{8,2,5,0,7,3,1,4,9,6},{2,9,0,1,8,6,3,7,5,4},

{4,9,7,6,0,5,2,1,8,3},{2,5,6,4,3,9,7,1,8,0},{1,8,7,6,3,9,2,5,4,0}

Time_Matrix:

{84,58,77,44,97,89,5,58,96,9},{21,87,15,39,81,85,31,57,73,77},

{40,71,34,82,70,22,10,80,48,49},{75,17,7,72,11,62,47,35,91,55},

{20,12,71,67,64,94,15,50,75,90},{93,93,57,70,77,58,52,29,7,68},

{56,95,48,26,82,63,36,27,87,6},{76,15,78,8,41,36,30,84,36,76},

{75,13,81,29,54,82,88,78,40,13},{6,26,32,64,54,52,82,6,88,54},

{62,67,32,62,69,61,35,72,5,93},{78,90,85,72,64,63,11,82,88,7},

{28,11,50,88,44,31,27,66,49,35},{14,39,56,62,97,66,69,7,47,76},

{18,93,58,47,69,57,41,53,79,64}

# LA24 15×10

Machine_Matrix:

{7,9,0,6,4,8,2,5,1,3},{6,8,3,0,1,4,5,9,2,7},{1,3,5,4,0,2,6,8,9,7},{1,7,4,6,5,0,8,3,9,2},

{7,2,8,5,1,6,3,0,9,4},{8,0,4,5,9,1,7,6,3,2},{6,2,8,1,9,4,7,0,5,3},{8,7,5,3,2,4,9,1,0,6},

{4,0,9,5,7,3,2,8,6,1},{9,0,3,8,1,6,2,5,4,7},{7,3,4,5,2,6,0,9,1,8},{0,3,2,7,8,5,9,1,6,4},

{9,1,3,6,2,8,7,0,5,4},{4,2,5,6,8,7,3,1,0,9},{2,5,9,8,0,6,3,7,1,4}

Time_Matrix:

{8,75,72,74,30,43,38,98,26,19},{19,73,43,23,85,39,13,26,67,9},

{50,93,80,7,55,61,57,72,42,46},{68,43,99,60,68,91,11,96,11,72},

{84,34,40,7,70,74,12,43,69,30},{60,49,59,72,63,69,99,45,27,9},

{71,91,65,90,98,8,50,75,37,17},{62,90,98,31,91,38,72,9,72,49},

{35,39,74,25,47,52,63,21,35,80},{58,5,50,52,88,20,68,24,53,57},

{99,91,33,19,18,38,24,35,49,9},{68,60,77,10,60,15,72,18,90,18},

{79,60,56,91,40,86,72,80,89,51},{10,92,23,46,40,72,6,23,95,34},

{24,29,49,55,47,77,77,8,28,48}

# LA25 15×10

Machine_Matrix:

{8,4,3,2,0,5,9,1,7,6},{5,3,2,4,6,9,0,1,7,8},{9,1,0,6,4,7,3,5,8,2},

{2,1,0,5,4,7,9,8,3,6},{6,2,3,8,4,7,1,5,9,0},{8,2,7,0,5,3,4,6,9,1},

{0,2,3,5,4,9,8,6,7,1},{3,7,9,0,2,4,5,1,8,6},{2,3,4,6,1,9,8,0,5,7},

{7,8,4,6,0,5,2,9,3,1},{8,6,7,4,5,3,0,2,9,1},{1,5,8,6,4,0,3,2,7,9},

{2,6,7,1,4,8,0,3,9,5},{6,2,5,8,1,7,9,4,3,0},{4,7,8,1,3,2,6,9,5,0}

Time_Matrix:

{14,75,12,38,76,97,12,29,44,66},{38,82,85,58,87,89,43,80,69,92},

{5,84,43,48,8,7,41,61,66,14},{42,8,96,19,59,97,73,43,74,41},

{55,70,75,42,37,23,48,5,38,7},{9,72,31,79,73,95,25,43,60,56},

{97,64,78,21,94,31,53,16,86,7},{86,85,63,61,65,30,32,33,44,59},

{44,16,11,45,30,84,93,60,61,90},{36,31,47,52,32,11,28,35,20,49},

{20,49,74,10,17,34,85,77,68,84},{85,7,71,59,76,17,29,17,48,13},

{15,87,11,39,39,43,19,32,16,64},{32,92,33,82,83,57,99,91,99,8},

{88,7,27,38,91,69,21,62,39,48}

# LA26 20×10

Machine_Matrix:

{8,7,6,9,2,1,5,4,0,3},{4,5,3,9,0,8,6,7,2,1},{5,4,2,6,1,7,0,3,9,8},{1,5,0,3,2,7,8,6,9,4},

{2,5,6,9,1,3,8,0,7,4},{1,4,0,2,9,8,5,3,7,6},{5,9,0,4,6,3,2,1,8,7},{5,9,8,7,4,6,3,0,1,2},

{1,8,0,2,9,3,5,6,4,7},{4,3,6,5,2,8,1,9,7,0},{4,7,9,2,3,8,5,6,1,0},{8,5,1,7,2,3,6,9,4,0},

{2,4,3,1,8,6,7,0,9,5},{0,8,3,7,5,2,4,6,1,9},{9,0,4,8,6,2,5,3,7,1},{3,2,5,0,7,4,8,1,6,9},

{1,7,8,3,4,5,6,0,2,9},{1,7,2,0,8,6,3,9,5,4},{2,3,4,9,0,6,7,8,1,5},{1,0,5,3,9,7,8,2,6,4}

Time_Matrix:

{52,26,71,16,34,21,95,21,53,55},{55,98,39,79,12,77,77,66,31,42},

{37,92,64,54,19,43,83,34,79,62},{87,77,93,69,87,38,24,41,83,60},

{98,25,75,77,49,17,79,44,43,96},{7,61,95,35,10,35,28,76,95,9},

{59,43,46,28,52,16,59,91,50,27},{9,43,14,71,20,54,41,87,45,39},

{28,66,78,37,42,26,33,89,33,8},{96,27,78,84,94,69,74,81,45,69},

{24,32,25,17,87,81,76,18,31,20},{90,28,72,86,23,99,76,97,45,58},

{17,98,48,46,27,67,62,42,48,27},{80,50,19,98,28,50,94,63,12,80},

{72,75,61,79,37,50,14,55,18,41},{96,14,57,47,65,75,79,71,60,22},

{31,47,58,32,44,58,34,33,69,51},{44,40,17,62,66,15,29,38,8,97},

{58,50,63,87,57,21,57,32,39,20},{85,84,56,61,15,70,30,90,67,20}

# LA27 20×10

Machine_Matrix:

{3,4,5,0,1,9,8,7,2,6},{7,6,0,5,2,3,4,9,8,1},{4,2,1,8,0,3,5,7,9,6},{0,2,9,1,8,3,6,5,7,4},

{4,2,9,7,5,6,0,3,8,1},{3,4,2,6,9,1,7,8,0,5},{5,6,4,0,1,7,2,3,9,8},{1,5,4,6,3,0,8,9,7,2},

{1,0,6,9,2,4,5,3,8,7},{3,2,7,1,8,0,6,5,9,4},{2,3,5,4,0,7,8,9,1,6},{4,7,1,8,0,3,2,5,9,6},

{9,6,4,3,1,0,8,2,7,5},{1,2,7,5,8,4,3,6,9,0},{6,1,3,0,2,8,4,7,9,5},{7,5,8,2,4,6,3,1,9,0},

{6,1,4,5,2,3,7,8,9,0},{0,5,8,9,3,6,4,7,2,1},{5,2,3,6,4,7,8,9,1,0},{9,4,6,7,0,2,8,5,3,1}

Time_Matrix:

{60,48,95,87,72,5,35,39,54,66},{37,34,97,55,21,20,59,46,19,46},

{45,73,24,28,28,25,23,83,5,78},{53,12,12,37,33,71,55,29,87,38},

{90,49,27,65,7,23,48,83,17,40},{85,25,84,64,13,66,46,59,62,19},

{88,67,14,41,73,57,53,80,47,74},{78,64,63,46,84,84,28,52,26,41},

{11,64,97,38,17,85,73,10,95,67},{93,95,43,65,32,59,85,46,85,60},

{61,41,49,23,66,49,70,99,90,17},{13,7,98,57,73,73,68,40,98,9},

{86,76,14,41,85,37,19,17,54,79},{40,53,97,87,96,84,16,66,52,95},

{33,33,87,18,55,13,77,60,42,74},{92,91,79,54,69,79,33,61,39,16},

{82,41,28,64,78,76,6,49,47,58},{52,42,24,91,47,88,91,52,28,35},

{82,76,86,93,84,38,95,37,21,33},{77,8,42,64,70,45,45,28,67,86}

# LA28 20×10

Machine_Matrix:

{8,1,4,7,0,5,9,3,2,6},{2,3,7,4,1,8,9,0,5,6},{7,4,3,1,2,8,9,6,5,0},

{4,5,0,2,6,3,1,7,9,8},{6,2,0,7,9,8,3,5,1,4},{7,9,0,6,4,8,2,5,1,3},

{6,8,3,0,1,4,5,9,2,7},{1,3,5,4,0,2,6,8,9,7},{1,7,4,6,5,0,8,3,9,2},

{7,2,8,5,1,6,3,0,9,4},{8,0,4,5,9,1,7,6,3,2},{6,2,8,1,9,4,7,0,5,3},

{8,7,5,3,2,4,9,1,0,6},{4,0,9,5,7,3,2,8,6,1},{9,0,3,8,1,6,2,5,4,7},

{7,3,4,5,2,6,0,9,1,8},{0,3,2,7,8,5,9,1,6,4},{9,1,3,6,2,8,7,0,5,4},

{4,2,5,6,8,7,3,1,0,9},{2,5,9,8,0,6,3,7,1,4}

Time_Matrix:

{32,81,55,40,6,19,81,37,40,9},{70,55,21,64,46,25,65,77,65,15},

{84,89,24,44,85,31,29,83,37,40},{80,59,8,30,77,38,80,56,41,97},

{40,71,91,7,59,80,50,56,17,88},{36,10,45,9,54,96,8,77,29,58},

{99,86,92,28,98,70,87,96,73,27},{95,85,56,52,59,41,81,39,32,92},

{7,69,93,27,22,88,45,60,49,12},{33,61,44,26,84,82,68,21,71,99},

{43,72,30,98,75,26,8,74,19,38},{19,67,73,85,26,39,9,23,13,43},

{72,46,80,93,61,7,42,50,55,57},{99,91,11,68,43,96,72,11,60,68},

{69,43,12,40,70,74,34,7,30,84},{99,27,59,72,9,45,49,63,69,60},

{75,17,91,50,65,37,98,90,71,8},{72,9,31,49,91,62,90,72,98,38},

{35,63,25,35,21,47,52,80,39,74},{68,24,58,52,5,20,50,57,88,53}

# LA29 20×10

Machine_Matrix:

{8,2,7,0,5,3,4,6,9,1},{0,2,3,5,4,9,8,6,7,1},{3,7,9,0,2,4,5,1,8,6},{2,3,4,6,1,9,8,0,5,7},

{7,8,4,6,0,5,2,9,3,1},{8,6,7,4,5,3,0,2,9,1},{1,5,8,6,4,0,3,2,7,9},{2,6,7,1,4,8,0,3,9,5},

{6,2,5,8,1,7,9,4,3,0},{4,7,8,1,3,2,6,9,5,0},{2,4,9,5,0,1,8,6,7,3},{0,5,1,3,2,4,6,8,9,7},

{6,4,8,7,2,3,5,0,1,9},{5,3,6,4,8,2,9,7,1,0},{3,5,2,8,7,6,1,9,4,0},{2,7,3,0,4,1,8,9,5,6},

{9,3,6,4,7,5,0,8,1,2},{3,2,6,8,1,7,0,5,9,4},{4,5,9,3,7,1,0,8,2,6},{5,2,0,7,8,4,3,6,9,1}

Time_Matrix:

{14,38,44,76,97,12,75,66,12,29},{43,85,82,38,58,89,92,87,69,80},

{41,7,5,43,14,8,61,84,66,48},{42,74,59,41,8,73,43,96,19,97},

{23,42,37,55,7,5,70,38,75,48},{9,43,31,25,73,95,79,72,60,56},

{7,21,53,16,94,97,78,64,86,31},{65,59,85,33,30,44,61,86,63,32},

{45,44,61,93,30,90,84,11,16,60},{47,36,31,49,20,28,52,35,11,32},

{77,10,68,17,85,84,20,49,74,34},{17,7,85,29,17,76,59,71,13,48},

{87,39,43,11,15,32,64,19,39,16},{33,99,32,91,82,92,99,57,83,8},

{91,39,69,27,7,21,38,62,88,48},{67,80,24,88,18,44,45,64,80,38},

{59,72,47,40,21,43,51,52,24,15},{70,31,20,76,40,43,32,88,5,77},

{47,64,85,49,58,26,32,80,14,94},{59,96,5,79,34,75,26,9,23,11}

# LA30 20×10

Machine_Matrix:

{6,3,1,8,7,4,9,0,5,2},{8,4,3,5,9,1,6,0,2,7},{3,2,7,5,4,0,8,9,6,1},

{4,5,2,8,1,0,6,7,3,9},{9,6,5,4,3,7,8,1,2,0},{1,8,2,6,9,0,3,7,5,4},

{5,6,3,9,2,0,7,8,4,1},{2,3,9,4,7,5,8,0,6,1},{3,4,6,0,1,2,9,5,7,8},

{0,2,4,7,3,5,6,8,9,1},{5,6,4,3,2,8,9,1,7,0},{0,1,2,7,4,9,8,3,6,5},

{2,9,0,5,4,6,1,8,3,7},{0,3,5,6,7,8,2,4,9,1},{1,5,8,2,6,3,4,9,7,0},

{1,5,4,7,2,8,9,3,6,0},{0,2,4,6,8,5,7,1,9,3},{5,2,1,4,0,3,7,6,9,8},

{2,8,4,6,5,9,1,3,7,0},{3,7,5,0,2,9,1,4,8,6}

Time_Matrix:

{32,16,33,12,70,10,75,82,88,20},{39,81,91,56,69,45,59,86,36,68},

{84,57,41,73,81,88,38,17,83,5},{20,6,15,19,30,94,45,17,18,88},

{24,49,16,11,60,5,63,25,15,45},{86,50,77,54,48,93,32,92,45,71},

{86,90,78,88,57,32,57,86,71,39},{59,18,31,41,20,83,65,54,94,69},

{47,79,76,59,72,8,30,73,57,84},{59,89,10,45,8,54,88,20,7,62},

{63,9,77,37,5,13,79,24,10,82},{74,32,61,53,92,20,10,5,45,23},

{85,51,61,99,37,94,98,65,33,75},{51,24,8,30,12,23,7,17,35,81},

{71,42,68,31,29,63,65,70,27,93},{28,38,51,70,33,78,45,90,54,72},

{18,90,25,92,85,35,29,81,80,59},{67,96,38,86,97,94,86,35,82,45},

{92,51,59,52,8,70,75,54,60,33},{98,80,78,82,7,89,69,51,79,62}

# LA31 30×10

Machine_Matrix:

{4,7,9,2,3,8,5,6,1,0},{8,5,1,7,2,3,6,9,4,0},{2,4,3,1,8,6,7,0,9,5},

{0,8,3,7,5,2,4,6,1,9},{9,0,4,8,6,2,5,3,7,1},{3,2,5,0,7,4,8,1,6,9},

{1,7,8,3,4,5,6,0,2,9},{1,7,2,0,8,6,3,9,5,4},{2,3,4,9,0,6,7,8,1,5},

{1,0,5,3,9,7,8,2,6,4},{5,7,6,0,3,2,9,4,1,8},{9,8,5,7,0,1,2,6,3,4},

{9,5,6,7,4,0,1,8,3,2},{9,3,5,1,4,6,7,8,0,2},{2,1,4,8,5,9,7,3,6,0},

{9,5,4,2,7,3,1,0,8,6},{3,2,4,1,9,0,6,5,7,8},{8,7,2,0,9,5,6,3,1,4}，

{3,2,6,4,7,8,5,9,0,1},{4,6,1,2,7,0,8,5,3,9},{6,0,4,3,7,8,1,5,2,9},

{3,9,6,5,0,8,4,2,7,1},{4,1,8,0,7,6,5,3,9,2},{9,1,4,3,8,2,6,0,7,5},

{3,2,6,9,7,0,4,5,1,8},{1,4,0,2,9,6,7,8,5,3},{1,3,0,2,9,7,8,4,6,5},

{5,3,6,1,0,7,8,9,2,4},{1,0,7,4,3,5,9,8,6,2},{4,8,2,3,1,6,7,9,5,0}

Time_Matrix:

{21,26,16,34,55,52,95,71,21,53},{77,98,42,66,31,39,77,79,55,12},

{64,92,34,19,62,54,43,83,79,37},{93,24,69,38,77,87,60,41,87,83},

{77,44,96,79,75,98,25,17,43,49},{76,35,28,95,95,61,35,7,9,10},

{91,27,50,16,28,59,52,46,59,43},{45,71,39,87,14,54,41,43,9,20},

{37,26,33,42,78,89,8,66,28,33},{74,69,84,27,81,45,69,94,78,96},

{76,32,18,20,87,17,25,24,31,81},{97,90,28,86,58,72,23,76,99,45},

{48,27,67,62,98,42,46,27,48,17},{80,19,28,12,94,63,98,50,80,50},

{50,41,61,79,14,72,18,55,37,75},{22,57,75,14,65,96,71,47,79,60},

{32,69,44,31,51,33,34,58,47,58},{66,40,17,62,38,8,15,29,44,97},

{50,58,21,63,57,32,20,87,57,39},{20,67,85,90,70,84,30,56,61,15},

{29,82,18,38,21,50,23,84,45,41},{54,37,62,16,52,57,54,38,74,52},

{79,61,11,81,89,89,57,68,81,30},{24,66,32,33,8,20,84,91,55,20},

{54,64,83,40,8,7,19,56,39,7},{6,74,63,64,15,42,98,61,40,91},

{80,75,26,87,22,39,24,75,44,6},{8,79,61,15,12,43,26,22,20,80},

{36,63,10,22,96,40,5,18,33,62},{8,15,64,95,96,38,18,23,64,89}

# LA32 30×10

Machine_Matrix:

{6,1,4,2,8,3,0,5,9,7},{7,2,9,4,1,5,8,0,3,6},

{2,5,0,3,1,6,4,8,7,9},{4,6,7,0,2,5,3,1,9,8},

{0,6,4,1,2,3,9,8,5,7},{2,6,3,5,1,8,0,9,4,7},

{4,3,1,5,6,7,8,9,0,2},{1,7,3,4,6,9,8,0,2,5},

{3,8,0,2,1,5,4,9,7,6},{0,2,3,5,6,9,8,4,7,1},

{8,1,4,7,0,5,9,3,2,6},{2,3,7,4,1,8,9,0,5,6},

{7,4,3,1,2,8,9,6,5,0},{4,5,0,2,6,3,1,7,9,8},

{6,2,0,7,9,8,3,5,1,4},{7,9,0,6,4,8,2,5,1,3},

{6,8,3,0,1,4,5,9,2,7},{1,3,5,4,0,2,6,8,9,7},

{1,7,4,6,5,0,8,3,9,2},{7,2,8,5,1,6,3,0,9,4},

{8,0,4,5,9,1,7,6,3,2},{6,2,8,1,9,4,7,0,5,3},

{8,7,5,3,2,4,9,1,0,6},{4,0,9,5,7,3,2,8,6,1},

{9,0,3,8,1,6,2,5,4,7},{7,3,4,5,2,6,0,9,1,8},

{0,3,2,7,8,5,9,1,6,4},{9,1,3,6,2,8,7,0,5,4},

{4,2,5,6,8,7,3,1,0,9},{2,5,9,8,0,6,3,7,1,4}

Time_Matrix:

{89,58,97,44,77,5,9,58,96,84},{31,81,73,15,87,39,57,77,85,21},

{48,71,40,70,49,22,10,34,80,82},{11,72,62,55,17,75,7,91,35,47},

{64,71,12,90,94,75,20,15,50,67},{29,93,68,93,57,77,52,7,58,70},

{26,27,63,6,87,56,48,36,95,82},{8,76,76,30,84,78,41,36,36,15},

{13,29,75,81,78,88,54,40,13,82},{52,6,6,82,64,88,54,54,32,26},

{62,35,72,69,62,32,5,61,67,93},{78,11,82,7,72,64,90,85,88,63},

{50,28,35,66,27,49,11,88,31,44},{62,39,76,14,56,97,7,69,66,47},

{47,41,64,58,57,93,69,53,18,79},{76,81,76,61,77,26,74,22,58,78},

{30,72,43,65,16,92,95,29,99,64},{35,74,16,85,7,81,86,61,35,34},

{97,43,72,88,17,43,94,64,22,42},{99,84,99,98,20,31,74,92,23,89},

{32,6,55,19,81,81,40,9,37,40},{15,70,25,46,65,64,21,77,65,55},

{31,84,37,24,85,89,29,44,40,83},{80,8,41,59,56,38,30,97,77,80},

{59,91,50,80,17,40,71,56,88,7},{36,58,54,77,8,9,45,10,29,96},

{28,92,73,27,86,87,96,98,99,70},{32,95,85,81,41,39,92,59,56,52},

{93,12,22,27,45,69,60,7,88,49},{61,26,71,44,21,82,68,33,84,99}

# LA33 30×10

Machine_Matrix:

{2,4,9,5,0,1,8,6,7,3},{0,5,1,3,2,4,6,8,9,7},

{6,4,8,7,2,3,5,0,1,9},{5,3,6,4,8,2,9,7,1,0},

{3,5,2,8,7,6,1,9,4,0},{2,7,3,0,4,1,8,9,5,6},

{9,3,6,4,7,5,0,8,1,2},{3,2,6,8,1,7,0,5,9,4},

{4,5,9,3,7,1,0,8,2,6},{5,2,0,7,8,4,3,6,9,1},

{5,3,6,1,0,8,7,9,4,2},{8,5,3,1,4,6,2,0,9,7},

{1,9,4,6,7,3,2,0,5,8},{5,8,2,1,6,3,9,4,0,7},

{7,0,9,4,6,5,8,3,1,2},{9,8,3,7,2,4,6,0,5,1},

{2,3,4,7,8,0,9,1,6,5},{4,7,1,2,8,6,5,3,9,0},

{2,7,9,5,1,6,0,3,8,4},{9,1,8,4,7,3,2,0,6,5},

{0,2,8,3,6,7,1,9,4,5},{8,1,4,7,6,0,2,9,3,5},

{4,9,3,0,6,8,2,7,5,1},{7,1,6,9,2,8,3,0,4,5},

{9,6,7,5,1,3,0,4,2,8},{7,2,1,5,9,6,0,3,8,4},

{1,8,7,5,4,9,2,6,0,3},{9,3,0,5,7,8,2,4,1,6},

{5,7,9,4,8,1,2,6,3,0},{2,6,8,9,4,7,1,0,3,5}

Time_Matrix:

{38,75,12,97,76,29,14,66,44,12},{43,38,80,82,85,58,87,92,89,69},

{48,8,66,7,14,41,61,43,84,5},{19,74,41,59,43,42,73,97,8,96},

{75,5,70,42,23,55,48,38,37,7},{72,31,95,79,25,56,9,60,73,43},

{31,78,16,94,86,21,97,53,7,64},{86,65,59,44,33,85,61,32,63,30},

{11,61,84,16,90,30,60,93,44,45},{11,28,32,36,31,47,20,52,35,49},

{17,34,49,84,85,20,74,68,10,77},{71,7,29,85,76,59,17,17,13,48},

{39,16,39,87,11,32,15,19,64,43},{33,82,92,83,32,99,99,91,8,57},

{7,48,62,88,21,39,27,91,38,69},{64,45,24,80,67,18,38,88,80,44},

{15,72,40,21,52,51,59,24,47,43},{77,43,40,31,76,20,88,70,5,32},

{14,58,85,64,26,94,32,49,80,47},{23,11,34,75,79,26,96,5,9,59},

{75,20,10,66,43,37,9,83,68,52},{54,26,79,88,84,6,54,59,28,42},

{56,29,36,40,86,68,69,23,62,16},{53,5,17,59,59,78,64,82,13,12},

{7,62,90,83,85,69,16,81,58,66},{24,65,69,42,82,82,83,46,72,33},

{10,27,43,20,71,65,73,99,24,64},{35,92,38,35,30,45,8,82,34,21},

{23,84,7,85,60,15,52,94,83,6},{70,29,27,80,6,39,79,28,66,66}

# LA34 30×10

Machine_Matrix:

{2,7,1,5,9,0,6,3,8,4},{4,7,5,2,8,0,3,6,9,1},

{6,1,2,3,9,0,4,5,8,7},{3,1,2,4,6,7,9,8,5,0},

{4,6,9,3,1,7,0,5,2,8},{4,3,6,9,0,8,5,2,1,7},

{0,8,9,4,6,3,5,1,2,7},{7,2,3,5,0,8,9,1,4,6},

{2,0,8,5,1,3,7,4,6,9},{7,9,3,4,0,1,6,8,2,5},

{4,6,0,8,3,1,2,9,5,7},{8,0,4,9,1,5,3,2,7,6},

{0,7,8,9,1,6,4,5,3,2},{6,1,7,5,8,9,0,3,4,2},

{0,1,7,5,3,4,2,9,8,6},{0,9,5,3,2,8,4,1,6,7},

{9,3,6,4,7,1,8,5,2,0},{1,3,7,0,6,2,8,9,5,4},

{8,7,2,4,1,9,5,6,3,0},{1,3,7,2,4,9,0,5,8,6},

{2,0,1,8,7,6,5,4,3,9},{1,8,5,4,6,2,3,0,7,9},

{4,6,7,0,1,2,8,3,9,5},{7,3,8,4,0,5,9,2,6,1},

{1,4,7,6,2,3,9,8,0,5},{9,5,2,8,4,6,7,0,1,3},

{7,0,6,2,8,9,1,4,3,5},{3,8,9,4,2,5,6,1,0,7},

{0,2,8,1,7,3,5,4,9,6},{3,6,2,1,0,5,9,7,4,8}

Time_Matrix:

{51,59,35,73,65,27,13,81,32,74},{64,33,75,33,10,28,38,53,49,55},

{83,23,72,7,72,6,39,52,90,21},{82,23,93,78,88,53,28,65,21,61},

{41,12,12,77,70,24,81,73,62,6},{98,28,42,72,15,15,94,33,51,99},

{32,22,96,15,78,31,7,94,23,86},{93,97,43,73,24,68,88,42,35,72},

{14,44,13,67,63,49,5,17,85,66},{82,15,72,26,8,68,21,45,99,27},

{93,23,51,54,49,96,56,36,53,52},{60,14,70,55,23,83,38,24,37,48},

{62,15,69,23,82,26,45,33,12,37},{72,9,15,28,92,12,59,64,87,73},

{50,14,90,46,71,48,80,61,24,44},{22,94,16,73,54,54,46,97,61,75},

{55,67,77,30,6,32,47,93,6,40},{30,98,79,22,79,7,36,36,9,92},

{37,72,52,31,82,54,7,82,73,49},{73,83,45,76,43,29,35,92,39,28},

{58,26,48,52,34,96,70,98,80,94},{70,23,26,14,90,93,21,42,18,36},

{28,76,25,17,84,67,87,43,88,84},{30,91,52,80,21,8,37,15,12,92},

{28,7,46,92,77,15,69,54,47,39},{50,44,64,38,93,33,75,41,24,5},

{94,17,87,21,92,28,61,63,34,77},{72,98,5,28,9,95,64,43,50,96},

{85,85,39,98,24,71,60,55,22,35},{78,49,46,11,90,20,34,6,70,74}

# LA35 30×10

Machine_Matrix:

{0,2,3,7,9,6,8,5,1,4},{3,0,6,2,8,9,1,4,7,5},

{4,3,8,9,1,6,5,2,7,0},{1,5,0,6,2,9,3,4,8,7},

{0,4,3,5,1,9,6,8,7,2},{7,0,1,6,5,3,4,2,8,9},

{7,5,1,8,2,4,3,0,9,6},{2,4,7,8,6,3,9,5,1,0},

{7,9,2,8,6,3,5,4,0,1},{9,6,5,1,2,8,0,4,3,7},

{8,0,9,3,6,5,4,2,7,1},{4,3,0,8,2,1,7,6,5,9},

{9,1,6,7,0,8,4,2,5,3},{0,6,1,3,8,5,4,9,7,2},

{2,0,5,6,3,7,1,9,4,8},{1,5,6,0,8,4,9,2,7,3},

{4,7,1,8,0,6,9,5,3,2},{1,0,7,8,4,5,9,2,3,6},

{0,6,1,5,9,8,3,4,2,7},{1,0,6,5,4,7,8,2,9,3},

{7,9,6,3,8,2,4,1,0,5},{2,5,4,0,6,9,8,7,3,1},

{4,3,6,2,9,0,7,8,5,1},{8,1,3,5,9,4,6,0,2,7},

{5,3,2,0,1,9,6,8,7,4},{7,2,0,9,5,6,1,4,3,8},

{5,0,9,2,1,6,7,8,3,4},{6,5,8,1,3,0,7,2,9,4},

{0,9,3,5,1,7,4,2,6,8},{3,9,2,0,6,8,4,5,1,7}

Time_Matrix:

{66,84,26,29,94,98,7,98,45,43},{32,97,55,88,93,88,20,50,17,5},

{43,68,47,68,57,20,81,60,94,62},{57,40,78,9,49,17,32,30,87,77},

{52,30,48,48,26,17,93,97,49,89},{95,33,5,17,70,57,34,61,62,39},

{97,92,31,5,79,5,67,5,78,60},{79,6,20,45,34,24,26,68,16,46},

{58,50,19,93,49,25,85,50,93,26},{81,71,7,39,16,42,71,84,56,99},

{9,86,6,71,97,85,16,42,81,81},{72,24,30,56,43,61,82,40,59,43},

{43,13,70,93,95,12,15,78,97,14},{14,26,71,46,80,31,37,27,92,67},

{12,43,96,7,45,20,13,29,60,33},{78,50,84,42,84,30,76,57,87,59},

{49,50,15,13,93,50,32,59,10,35},{25,47,60,33,53,37,73,22,87,79},

{84,83,71,68,89,11,60,50,33,97},{14,38,88,5,77,92,24,73,52,71},

{62,19,38,15,64,64,8,61,19,33},{33,46,74,56,84,83,19,8,32,97},

{50,71,50,97,8,17,19,92,54,52},{32,79,97,38,49,76,76,56,78,54},

{13,5,25,86,95,28,78,24,10,39},{48,59,20,7,31,97,89,32,25,41},

{87,18,48,43,30,97,47,65,69,27},{71,20,20,78,39,17,50,44,42,38},

{50,42,72,7,77,58,78,89,70,36},{32,95,13,73,97,24,49,57,68,94}

# LA36 15×15

Machine_Matrix:

{4,3,6,14,10,2,9,1,0,7,8,5,12,11,13},{11,4,1,7,8,14,12,0,3,6,9,5,10,13,2},

{9,5,2,7,4,12,0,13,6,10,3,11,8,1,14},{5,0,9,6,4,13,7,8,11,12,2,1,10,14,3},

{10,4,8,2,0,11,14,9,6,7,1,13,3,5,12},{0,2,4,13,3,12,14,6,1,9,11,8,7,10,5},

{8,4,12,0,7,11,6,10,3,13,1,5,14,2,9},{4,13,11,3,7,9,1,2,12,8,14,0,10,6,5},

{5,1,6,8,13,10,2,3,7,11,14,4,0,9,12},{11,5,4,8,7,0,9,6,14,3,10,13,2,12,1},

{7,3,0,4,12,14,10,1,9,13,5,8,2,11,6},{1,2,3,5,4,6,9,7,10,8,11,13,12,0,14},

{3,7,13,5,11,12,2,4,10,1,9,6,14,8,0},{9,7,5,14,10,4,11,2,1,3,13,6,0,12,8},

{9,10,11,14,8,0,7,6,12,1,2,13,4,3,5}

Time_Matrix:

{21,55,71,98,12,34,16,21,53,26,52,95,31,42,39},

{54,83,77,64,34,79,43,55,77,19,37,79,92,62,66},

{83,77,87,38,60,98,93,17,41,44,69,49,24,87,25},

{77,96,28,7,95,35,35,76,9,95,43,75,61,10,79},

{87,28,50,59,46,45,9,43,52,27,91,41,16,59,39},

{20,71,78,66,14,8,42,28,54,33,89,26,37,33,43},

{69,96,17,69,45,31,78,20,27,87,74,84,76,94,81},

{58,90,76,81,23,28,18,32,86,99,97,24,45,72,25},

{27,46,67,27,19,80,17,48,62,12,28,98,42,48,50},

{37,80,75,55,50,94,14,41,72,50,61,79,98,18,63},

{65,96,47,75,69,58,33,71,22,32,57,79,14,31,60},

{34,47,58,51,62,44,8,17,97,29,15,66,40,44,38},

{50,57,61,20,85,90,58,63,84,39,87,21,56,32,57},

{84,45,15,41,18,82,29,70,67,30,50,23,20,21,38},

{37,81,61,57,57,52,74,62,30,52,38,68,54,54,16}

# LA37 15×15

Machine_Matrix:

{5,6,11,9,2,14,3,10,12,13,1,7,8,4,0},{1,3,7,2,14,0,9,8,6,5,13,4,10,12,11},

{6,8,0,9,10,12,2,11,13,3,5,1,4,14,7},{10,12,3,4,2,0,8,7,13,9,11,6,1,14,5},

{9,8,3,6,0,7,1,4,13,5,11,10,2,14,12},{11,7,5,6,0,3,13,4,2,10,12,8,14,1,9},

{4,2,9,5,14,13,10,12,0,3,7,8,6,1,11},{6,5,11,9,14,10,7,13,1,4,2,3,12,0,8},

{11,5,9,2,0,6,12,13,10,1,7,4,3,14,8},{11,1,3,13,9,14,0,10,7,4,5,6,12,8,2},

{4,12,1,7,5,0,11,3,2,13,6,10,9,14,8},{12,1,10,5,8,6,2,13,4,11,0,14,3,9,7},

{2,7,5,9,1,14,3,10,13,12,0,6,8,4,11},{11,6,1,12,9,5,3,13,10,0,2,8,14,7,4},

{13,3,4,9,11,10,0,8,2,6,5,1,7,12,14}

Time_Matrix:

{19,64,73,13,84,88,85,41,53,80,66,46,59,25,62},

{67,74,41,57,52,14,64,84,78,47,28,84,63,26,46},

{97,95,64,38,59,95,17,65,93,10,73,11,85,46,67},

{23,49,32,66,43,60,41,61,70,49,17,90,85,99,85},

{98,57,73,9,73,7,98,13,41,40,85,37,68,79,17},

{66,53,86,40,14,19,96,95,54,84,97,16,52,76,87},

{77,55,42,74,91,33,16,54,18,87,60,13,33,33,61},

{41,39,82,64,47,28,78,49,79,58,92,79,6,69,76},

{21,42,91,28,52,88,76,86,23,35,52,91,47,82,24},

{42,93,95,45,28,77,84,8,45,70,37,86,64,67,38},

{97,81,58,84,58,9,87,5,44,85,89,77,96,39,77},

{80,21,10,73,70,49,31,34,40,22,15,82,57,71,48},

{17,62,75,35,91,50,7,64,75,94,55,72,47,11,90},

{93,57,71,70,93,20,15,77,58,12,67,68,7,29,52},

{76,27,26,36,8,36,95,48,82,87,6,63,56,36,15}

# LA38 15×15

Machine_Matrix:

{1,12,0,6,14,8,4,3,10,11,5,13,2,7,9},{14,0,4,12,1,8,9,5,10,6,11,3,2,7,13},

{3,4,12,6,7,14,13,8,11,10,0,1,9,5,2},{14,11,4,1,8,0,9,7,12,13,5,6,2,3,10},

{2,0,9,3,10,13,11,14,7,5,4,8,1,12,6},{11,6,4,3,14,5,10,9,1,8,7,2,13,12,0},

{14,7,10,13,3,0,11,12,9,5,8,1,2,6,4},{7,14,5,11,4,6,3,0,8,10,12,13,1,2,9},

{13,8,2,9,12,11,5,10,6,7,0,1,3,4,14},{1,6,5,11,4,0,14,8,10,7,12,2,13,9,3},

{10,4,12,0,9,1,11,6,5,8,3,7,14,2,13},{5,14,2,3,13,6,1,7,4,8,12,11,10,9,0},

{9,14,3,8,2,6,1,4,5,0,13,10,12,11,7},{10,3,4,5,11,1,8,2,13,0,7,12,6,14,9},

{3,5,11,7,14,9,6,10,0,1,2,8,13,12,4}

Time_Matrix:

{26,67,72,74,13,43,30,19,23,85,98,43,38,8,75},

{42,39,55,46,19,93,80,26,7,50,57,73,9,61,72},

{96,99,34,60,43,7,12,11,70,43,91,68,11,68,72},

{63,45,49,74,27,30,72,9,99,60,69,69,84,40,59},

{91,75,98,17,72,31,9,98,50,37,8,65,90,91,71},

{35,80,39,62,74,72,35,25,49,52,63,90,21,47,38},

{19,57,24,91,50,5,49,18,58,24,52,88,68,20,53},

{77,72,35,90,68,18,9,33,60,18,10,60,38,99,15},

{6,86,40,79,92,23,89,95,91,72,80,60,56,51,23},

{46,28,34,77,47,10,49,77,48,24,8,72,55,29,40},

{22,89,79,7,15,6,30,38,11,52,20,5,9,20,28},

{73,56,37,22,25,58,8,93,88,17,9,69,71,85,55},

{85,58,46,64,49,37,33,30,26,20,74,77,99,56,21},

{17,24,89,15,60,42,98,64,92,63,52,54,75,23,38},

{8,17,56,93,26,62,7,88,97,7,43,29,35,87,57}

# LA39 15×15

Machine_Matrix:

{10,14,7,4,6,3,2,12,11,13,8,5,9,1,0},

{6,9,10,5,11,3,8,0,4,13,12,7,2,14,1},

{0,3,10,5,7,8,6,11,1,13,14,9,4,12,2},

{5,6,0,12,11,2,4,3,7,13,14,10,9,8,1},

{12,9,3,14,13,1,6,11,8,5,2,0,10,7,4},

{13,12,11,5,4,2,9,8,1,7,6,3,14,10,0},

{10,13,9,12,11,6,0,2,8,5,7,14,4,3,1},

{7,10,8,6,9,11,4,14,5,3,12,1,13,2,0},

{4,1,3,2,0,8,6,5,12,9,11,14,10,13,7},

{2,5,9,10,4,13,6,7,0,1,3,14,12,8,11},

{3,6,13,8,1,5,12,4,9,0,11,7,14,10,2},

{14,10,5,11,12,0,9,6,2,1,8,7,4,13,3},

{7,0,4,5,9,10,12,13,8,6,11,14,3,2,1},

{3,4,0,2,10,12,8,5,9,7,6,11,13,1,14},

{3,7,0,1,10,12,5,6,14,9,8,13,11,2,4}

Time_Matrix:

{51,43,80,18,38,24,67,15,24,72,45,80,64,44,88},

{40,88,77,59,20,52,70,40,32,76,43,31,21,5,47},

{32,49,5,64,58,80,94,11,26,26,59,85,47,96,14},

{23,9,75,37,43,79,75,34,20,10,83,68,52,66,9},

{69,59,28,62,36,26,84,16,54,42,54,6,40,88,79},

{78,53,17,29,82,23,12,64,86,59,5,68,59,13,56},

{83,46,7,65,69,62,16,58,66,83,90,42,81,69,85},

{73,71,64,10,20,99,24,65,82,72,43,82,27,24,33},

{82,34,92,8,38,45,21,35,52,35,15,23,6,83,30},

{84,7,66,6,28,27,79,70,85,94,60,80,39,66,29},

{44,58,14,65,72,14,52,21,25,5,51,61,55,42,36},

{43,72,78,12,17,46,27,51,63,79,79,91,49,26,93},

{49,49,71,78,44,41,91,84,91,21,47,28,61,70,93},

{25,85,66,45,95,21,84,24,53,67,91,11,32,30,89},

{92,93,99,40,37,69,66,57,22,44,73,97,18,69,41}

# LA40 15×15

Machine_Matrix:

{9,10,4,12,2,14,5,8,6,3,1,7,13,11,0},{0,1,11,2,4,9,14,8,13,12,6,3,10,5,7},

{14,3,1,12,6,5,8,11,7,10,2,13,0,9,4},{1,6,7,4,14,10,9,5,11,2,13,8,3,12,0},

{12,5,9,4,14,13,0,8,11,1,2,7,6,10,3},{1,5,2,13,4,14,6,7,9,10,11,0,3,12,8},

{9,7,6,14,3,13,2,4,12,8,1,10,0,5,11},{3,7,4,8,5,2,14,12,11,0,13,10,1,6,9},

{3,8,6,9,14,1,5,4,13,7,11,12,10,2,0},{0,5,7,4,10,12,1,13,6,8,11,9,2,14,3},

{2,0,6,4,3,5,12,9,14,13,8,7,11,10,1},{10,14,4,9,3,1,12,13,6,8,11,7,5,2,0},

{0,13,3,6,1,14,11,4,10,9,5,8,7,2,12},{10,12,13,4,1,3,8,5,9,0,6,7,2,14,11},

{1,10,6,12,4,8,3,7,13,11,5,9,2,14,0}

Time_Matrix:

{65,28,74,33,51,75,73,32,13,81,35,59,38,55,27},

{64,53,83,33,6,52,72,7,90,21,23,10,39,49,72},

{73,82,23,62,88,21,65,70,53,81,93,77,61,28,78},

{12,51,33,15,72,98,94,12,42,24,15,28,6,99,41},

{97,7,96,15,73,43,32,22,42,94,23,86,78,24,31},

{72,88,93,13,44,66,63,14,67,17,85,35,68,5,49},

{15,82,21,53,72,49,99,26,56,45,68,51,8,27,96},

{54,24,14,38,36,52,55,37,48,93,60,70,23,23,83},

{12,69,26,23,28,82,33,45,64,15,9,73,59,37,62},

{87,12,80,50,48,90,72,24,14,71,44,46,15,61,92},

{54,22,61,46,73,16,6,94,93,67,54,75,32,40,97},

{92,36,22,9,47,77,79,36,30,98,79,7,55,6,30},

{49,83,73,82,82,92,73,31,35,54,7,37,72,52,76},

{98,34,52,26,28,39,80,29,70,43,48,58,45,94,96},

{70,17,90,67,14,23,21,18,43,84,26,36,93,84,42}
